# Long-term spatial–temporal evolution of hepatitis C burden in eastern China: a retrospective case analysis from Wuxi City

**DOI:** 10.3389/fpubh.2025.1665521

**Published:** 2025-11-25

**Authors:** Huan Ding, Qi Zhang, Shiya Shen, Song Gao, Shipeng Zhang, Zhuping Xu

**Affiliations:** Department of Chronic Infectious Disease Control and Prevention, The Affiliated Wuxi Center for Disease Control and Prevention of Nanjing Medical University, Wuxi Center for Disease Control and Prevention, Wuxi, China

**Keywords:** hepatitis C virus, epidemiology, spatiotemporal analysis, genotype distribution, public health surveillance, China

## Abstract

**Background:**

Hepatitis C is an inflammation of the liver caused by the hepatitis C virus (HCV) and represents a major public health challenge. This study aimed to explore the epidemiological characteristics and spatiotemporal distribution of hepatitis C in Wuxi City, a developed and low-epidemic area of eastern China, from 2007 to 2021.

**Methods:**

Data were obtained from the Chinese Information System for Disease Control and Prevention, while genotype information was derived from a retrospective survey. Statistical analyses were conducted using SPSS software to identify characteristic differences. Joinpoint regression analysis was applied to examine long-term temporal trends, and the concentration degree was used to quantify seasonal intensity. Kernel density and hotspot analyses were applied to identify spatial distribution patterns and clusters.

**Results:**

A total of 4,771 cases were recorded, with an average annual incidence rate of 5.44 per 100,000 population. The incidence was significantly higher in males than in females (odds ratio = 1.49, *χ*^2^ = 144.58, *p* < 0.05). The incidence of HCV infection increased progressively with age. Genotype 1b (74.5%) was the most prevalent, followed by genotype 3 (15.2%), with subtype 3b being dominant. Sex and age were significantly associated with variations in genotype distribution (both *p* < 0.001). During the study period, the incidence rate increased from 2.18 to 9.15 per 100,000 population, with an average annual percentage change of 10.50% (95% confidence interval: 6.42–14.74%). No distinct seasonal pattern was observed, with a concentration degree (*M*) of 0.095. Spatially, HCV cases were mainly concentrated in the city center, and the hotspot areas showed little change across the three study periods.

**Conclusion:**

The incidence of HCV in Wuxi City has increased steadily from 2007 to 2021, with genotypes 1b and 3 being predominant. Spatial clustering was mainly concentrated in urban centers, highlighting the need for targeted prevention and control strategies to support the goal of eliminating HCV as a public health threat by 2030.

## 1 Introduction

Hepatitis C virus (HCV) is a bloodborne virus pathogen that poses major public health challenges due to its unfavorable long-term clinical outcomes, including chronic hepatitis, liver cirrhosis, and hepatocellular carcinoma (1). In 2016, the World Health Organization (WHO) launched the *Global Health Sector Strategy for Viral Hepatitis*, which was renewed in 2022, with the aim of eliminating hepatitis B and C as public health threats by 2030. Nevertheless, as of 2019, an estimated 58 million people live with chronic HCV infection, resulting in approximately 399,000 deaths worldwide (2).

Although the overall prevalence of hepatitis C in China is relatively low, the country has the largest number of patients globally. Approximately 7.6 million individuals are chronically infected with HCV (3), and the incidence continues to increase (4). Because HCV infection is often asymptomatic, many infected individuals are unaware of their condition (5). Consequently, the true prevalence of chronic HCV infection is likely underestimated.

The global prevalence of HCV infection varies considerably across WHO regions. The Eastern Mediterranean Region reports the highest prevalence (2.3%), while the Southeast Asia Region reports the lowest (0.5%). The Western Pacific Region, which includes China, has an intermediate prevalence of 0.7% (6). Consistent with the global pattern, studies have demonstrated substantial geographical disparities within China. For instance, using the Yangtze River as a boundary, the prevalence in the northern region (0.53%) is higher than that in the southern region (0.29%) (3).

Given the marked genetic diversity of HCV, the virus can be classified into genotypes, subtypes, strains, and quasispecies, contributing further to regional heterogeneity (7). To date, at least eight genotypes and more than 90 subtypes have been identified. Genotypes 1, 2, and 3 are the most widely distributed globally, whereas genotypes 4 and 5 predominate in Africa and the Middle East, and genotype 6 is primarily found in Southeast Asia. In contrast, genotypes 7 and 8 are rare and have been detected in only a few individuals (8). In China, subtypes 1b and 2a are most prevalent, with subtype 1b being the dominant strain (9–11). Notably, the geographic distribution of HCV genotypes serves as an important epidemiological marker for tracing infection sources within specific populations (12).

Spatiotemporal analysis provides a robust approach for tracing the transmission routes, predicting epidemic trends, evaluating outbreak risks, and formulating prevention and control strategies for HCV infection. By analyzing the temporal trends and spatial distributions, researchers can better understand the epidemic patterns and prevention requirements of hepatitis C and identify priority areas for intervention. Several studies employing spatial–temporal analysis (13, 14) have revealed that hotspots shifted gradually from northeast to western China between 2003 and 2015. These hotspots were predominantly concentrated in economically underdeveloped or border regions, with rural areas showing higher incidence rates than urban areas. However, Yang et al. (15) reported that the urbanization rate was the most critical factor influencing HCV incidence in Jiangsu Province, and that hotspots were progressively shifting from northern to southern regions of the Province.

Wuxi City is one of the 13 prefecture-level cities in Jiangsu Province. However, the spatiotemporal patterns and genotype distribution of HCV in Wuxi remain unclear. Key questions, such as whether spatial clustering exists and where hotspot areas are located, have not been answered. Therefore, this study aimed to address these gaps by conducting a comprehensive spatiotemporal analysis of HCV infection. The findings may enhance understanding of hepatitis C epidemiology and provide valuable insights for more effective prevention and control strategies.

## 2 Methods

### 2.1 Study area

**Figure 1:**
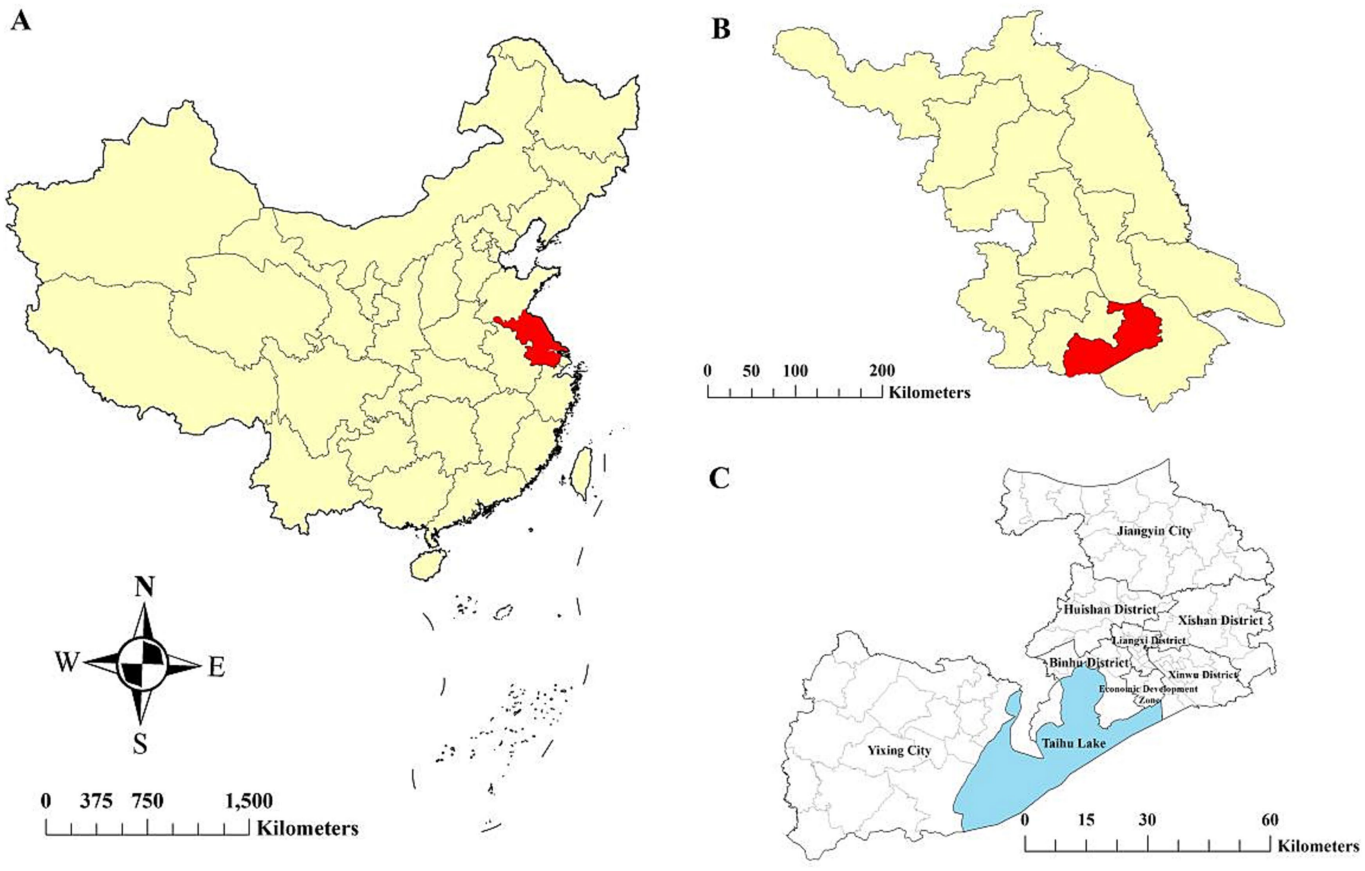
Location of the study area. **(A)** Jiangsu Province in China, **(B)** Wuxi City in Jiangsu Province, and **(C)** six districts and two county-level cities within Wuxi City.

Wuxi City, located in southern Jiangsu Province, lies between the Yangtze River to the north and Taihu Lake to the south (Figure 1). It covers an area of 4627.5 km^2^ and consists of six municipal districts and two county-level municipalities (Jiangyin and Yixing), with a total permanent population of 7.46 million. Wuxi has a highly developed economy and ranked among the top Chinese cities in 2020, with a per capita gross domestic product (GDP) exceeding $ 20,000 (16).

### 2.2 Criteria for case diagnosis

According to the *Diagnostic Criteria for Hepatitis C (WS 213–2018)* (17), Hepatitis C cases include confirmed and clinically diagnosed cases. Confirmed cases are defined as those with detectable HCV RNA in the blood. Clinically diagnosed cases are defined as anti-HCV positive individuals who meet any of the following criteria: (a) epidemiological history of exposure; (b) clinical manifestations consistent with hepatitis C; and (c) abnormal biochemical test results.

### 2.3 Data sources

Data on registered HCV cases from 2007 to 2021 were obtained from the Chinese Information System for Disease Control and Prevention. The analysis was conducted based on the current residential addresses of reported cases. Genotype data were acquired from a 2021 survey of previously reported HCV cases in Wuxi City. Demographic data were sourced from the *Wuxi City Statistical Yearbook*.

### 2.4 Data analysis

#### 2.4.1 Descriptive analysis

Descriptive analysis was performed to characterize the demographic distribution of HCV infection. Using SPSS software (version 20.0), we applied the Pearson Chi-square test, the linear-by-linear association test, or Fisher’s exact test to assess group differences. Statistical significance was defined as *p* < 0.05, and odds ratios (ORs) were calculated to quantify associations.

#### 2.4.2 Temporal analysis

##### 2.4.2.1 Joinpoint regression

A joinpoint regression model (18) was employed to examine the long-term temporal trend in HCV incidence in Wuxi from 2007 to 2021. In this model, crude HCV incidence rates served as the dependent variable, and calendar year was the independent variable. The best-fit model was selected based on the Bayesian information criterion, calculated using a k-joinpoint model reported by Zhang et al. (19). The annual percentage change, average annual percentage change, and 95% confidence intervals (CIs) were calculated as primary outcome indicators. Analyses were conducted using the Joinpoint Regression Program (version 4.9.1.0; US National Cancer Institute Surveillance Research Program).

##### 2.4.2.2 Concentration degree

The concentration degree (20) was used to assess the seasonal intensity of HCV onset, reflecting the degree of temporal aggregation of the disease within a year. The concentration degree value (M) was calculated from the ratio of the number of cases in each month to the total number of cases in the entire year. The formula was as follows:


$$M=\sqrt{R_{x}^{2}+R_{y}^{2}}$$

$$R_{x}=\frac{1}{2}(r_{2}+r_{6}-r_{8}-r_{12})+\frac{\sqrt{3}}{2}(r_{3}+r_{5}-r_{9}-r_{11})+(r_{4}-r_{10})$$

$$R_{y}=\frac{1}{2}(r_{3}-r_{5}-r_{9}+r_{11})+\frac{\sqrt{3}}{2}(r_{2}-r_{6}-r_{8}+r_{12})+(r_{1}-r_{11})$$

Where *M* represents the concentration degree, *R_i_* denotes the dispersion degree, *and r_i_* is the ratio of the number of cases in month *i* to the total number of cases in the year. The value of *M* ranges from 0 to 1, with *M* > 0.90 indicating a distinct seasonal pattern, 0.90 ≥ *M* > 0.50 suggesting a strong seasonal trend, 0.50 ≥ *M* > 0.30 implying a moderate seasonality, and *M* ≤ 0.30 reflecting a relatively uniform distribution with weak seasonality.

#### 2.4.3 Spatial analysis

Kernel density estimation was employed to analyze the spatial distribution of HCV cases. Kernel density estimation treats each sample point as a central nucleus and calculates the density per unit area within a defined search radius (bandwidth) to represent the spatial concentration of geographic elements (21). In a given region, a higher number of occurrences of a particular event indicates a greater spatial frequency of that event, whereas a lower count reflects a lower spatial frequency. The density was calculated using the following formula:


$$f_{\left(x,y\right)}=\frac{1}{nh^{2}}\sum_{i=1}^{n}k(\frac{d_{i,(x,y)}}{h})$$

Where *f*_(*x*, *y*)_ represents the density value at the spatial coordinate position (*x*, *y*), *n* is the number of HCV cases within the distance scale, *h* denotes the bandwidth, *k* is the kernel density function, and *d*_*i*(*x*, *y*)_ represents the distance from HCV case *i* to (*x*, *y*). ArcGIS software (version 10.4; ESRI) was used for kernel density analysis of the HCV spatial distribution characteristics in Wuxi City.

#### 2.4.4 Cluster analysis

Hot spot analysis using the Getis-Ord Gi* statistic (22) was performed to detect spatial clustering patterns, identifying statistically significant hot and cold spots. HCV case data were first aggregated by sub-districts, and incidence rates were calculated. The Getis-Ord Gi* statistic was then computed for each subdistrict based on the equation reported by Zhang et al. (19). Statistical inference was performed using the z-test: values of Gi* > 0 and *z* > 1.96 indicated spatial clustering of high incidence (hot spots), while Gi* < 0 and *z* < −1.96 indicated clustering of low incidence (cold spots). A *z*-score near 0 indicated no spatial clustering. The hot spot analyses were conducted using ArcGIS software (version 10.4; ESRI), with 999 random permutations to assess statistical significance at 95% CIs.

## 3 Results

### 3.1 Population distribution

As shown in Table 1, a total of 4,771 cases of HCV were reported from 2007 to 2021 in Wuxi City, corresponding to an average annual incidence rate of 5.44 per 100,000 population. Among these cases, 2,838 were males, with a male-to-female ratio of 1.47:1. The incidence rate was significantly higher in males than in females (OR = 1.49, *χ*^2^ = 144.58, *p* < 0.05). The incidence of HCV infection increased constantly with age. Using the preceding age group as the reference category, the OR values for the 20–39, 40–59, and ≥60 age groups were 8.24, 2.04, and 1.57, respectively (all *p* < 0.001). Although individuals aged 40–59 accounted for the largest proportion of HCV cases over the 15-year period (43.7%, 2087/4771), the incidence rate was highest among those aged ≥60, reaching 9.81 per 100,000 population. Among all reported cases, confirmed cases accounted for 80.59% (3603/4471), and males had a significantly higher incidence rate than females within this subgroup (OR = 1.52, *χ*^2^ = 103.03, *p* < 0.05).

**Table 1:** The number and incidence of HCV cases with different characteristics in Wuxi City from 2007 to 2021.

Characteristics	Males		Females		Total	
	Cases, *n*	Incidence*	Cases, *n*	Incidence*	Cases, *n*	Incidence*
*Age group (years)*						
0–19	51	0.51	17	0.20	68	0.37
20–39	577	3.40	426	2.69	1003	3.05
40–59	1320	7.68	767	4.70	2087	6.23
≥60	890	11.09	723	8.59	1613	9.81
*Case classification*						
Confirmed cases	2170	4.46	1433	2.92	3603	4.38
Clinically diagnosed cases	668	1.38	500	1.02	1168	1.06
Total	2838	5.84	1933	3.93	4,771	5.44

*Average annual incidence rate per 100,000 people.

### 3.2 Genotype distribution

Based on the cumulative number of reported HCV cases, the study area (six municipal districts and two county-level municipalities) was stratified into high-, medium-, and low-prevalence categories. From each stratum, 600, 400, and 200 cases were randomly sampled, respectively, yielding 3,000 cases. After excluding individuals who could not be contacted, had died, or no longer resided locally, 1,013 patients were included in the final analysis. Among these, 288 were nucleic acid-positive, and genotyping results were available for 243 cases.

As shown in Table 2, genotype 1b was the most common (74.5%), followed by genotype 3 (15.2%), with subtype 3b being predominant. Genotypes 2a and 6 accounted for 6.6 and 3.7%, respectively. The distribution of HCV genotypes was significantly influenced by sex and age (both *p* < 0.001). Among reasons for HCV antibody testing, the proportion of high-risk individuals undergoing passive screening was higher than that of those tested after developing clinical symptoms. However, the difference was not statistically significant (*p* = 0.735).

**Table 2:** The genotype distribution and influencing factors of HCV cases in Wuxi City, 2007–2021.

Factors	HCV genotypes				Fisher’s exact test
	1b (*n* = 181)	2a (*n* = 16)	3 (*n* = 37)	6 (*n* = 9)	
Gender					*P* < 0.001
Male	81	7	32	5	
Female	100	9	5	4	
Age group (years)					*P* < 0.001
0–19	17	1	7	1	
20–39	32	1	9	2	
40–59	68	9	20	6	
≥60	64	5	1	0	
Reasons for HCV antibody testing					*P* = 0.735
High-risk population passive screening and others	138	11	29	6	
Showed clinical symptoms and liver function	43	5	8	3	

### 3.3 Temporal distribution

**Figure 2:**
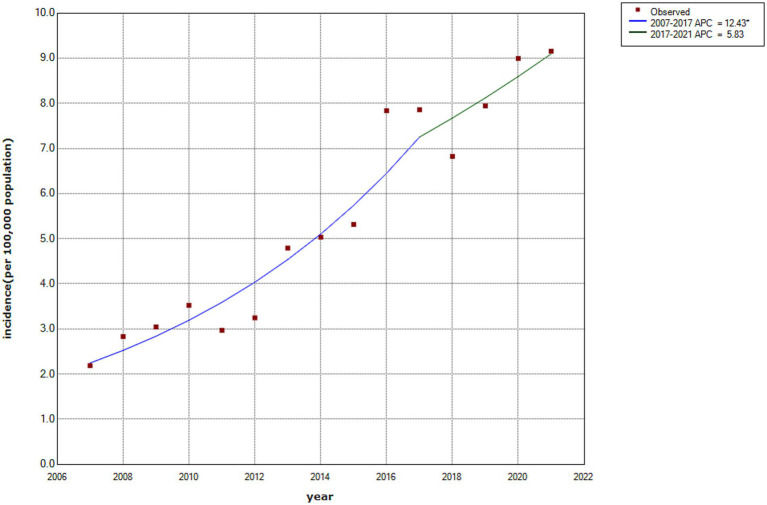
Temporal trend of HCV incidence in Wuxi City, 2007–2021.

As illustrated in Figure 2, the incidence rate of HCV infection in Wuxi City increased from 2.18 to 9.15 per 100,000 population between 2007 and 2021, with an average annual percentage change of 10.50% (95% CI: 6.42–14.74%). Joinpoint regression analysis identified two distinct phases. From 2007 onward, the incidence increased annually, with an annual percentage change of 12.43% (95% CI: 8.59–16.02%), reaching a peak during 2016–2017. Subsequently, the rate continued to increase gradually, with an annual percentage change of 5.83% (95% CI: −0.7–15.9%) from 2017 to 2021. Although a marked decline was observed in 2018, the joinpoint trend analysis did not reveal a statistically significant downward trend during this period.

**Figure 3:**
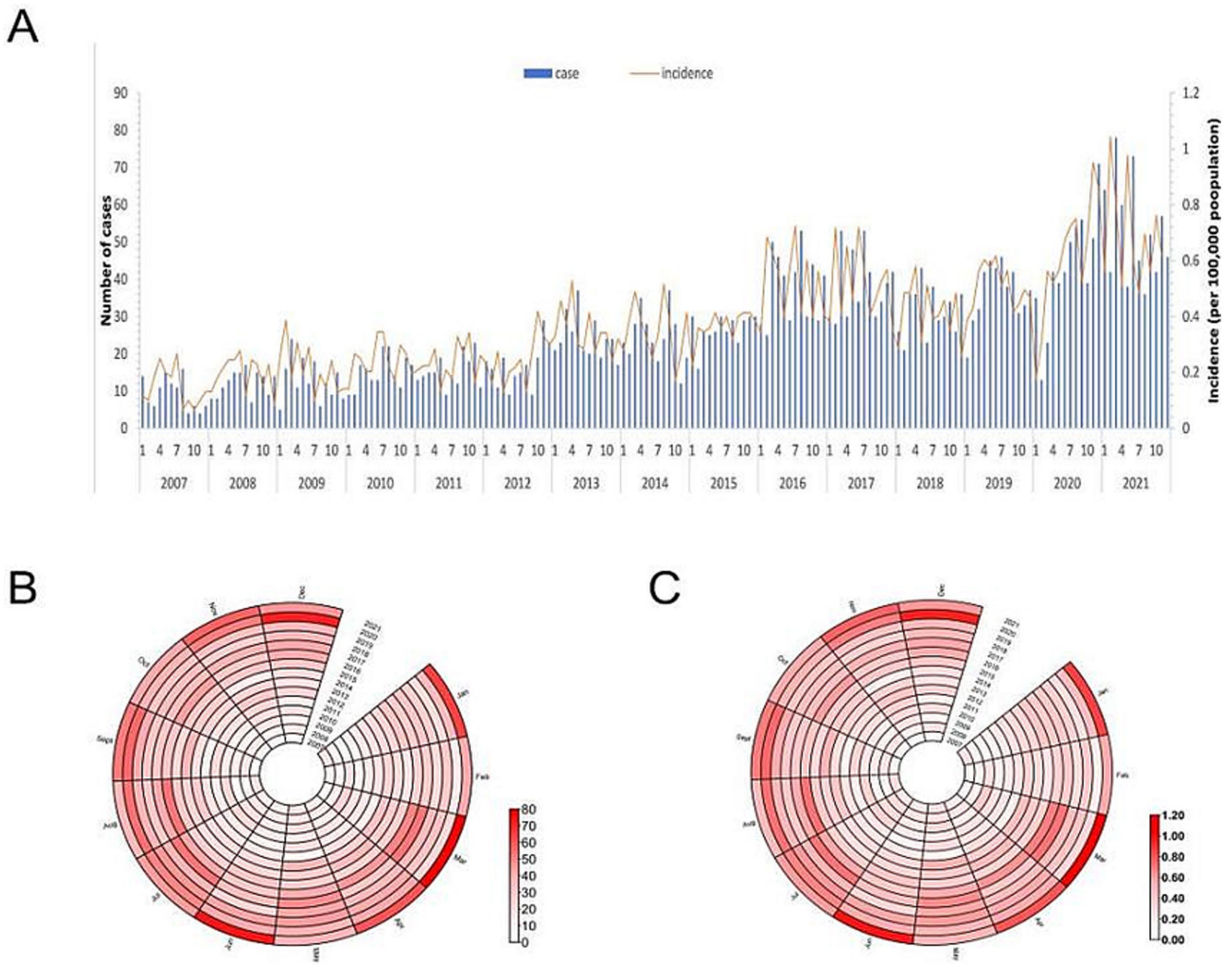
**(A)** Number and the incidence of HCV cases reported each month, **(B)** Monthly distribution of HCV cases, and **(C)** Monthly distribution of HCV incidence in Wuxi city, 2007-2021.

Monthly reporting from 2007 to 2021 showed the fewest HCV cases in February. As shown in Figure 3A, the seasonal trend exhibited no clear seasonal pattern and presented a multimodal distribution. The overall *M* value, calculated using the formula described earlier, was 0.095 (95% CI: 0.071–0.117). In contrast, annual *M* values ranged from 0.037 to 0.185, indicating weak seasonal intensity in HCV cases (Figure 3B) and incidence (Figure 3C).

### 3.4 Spatial distribution

**Figure 4:**
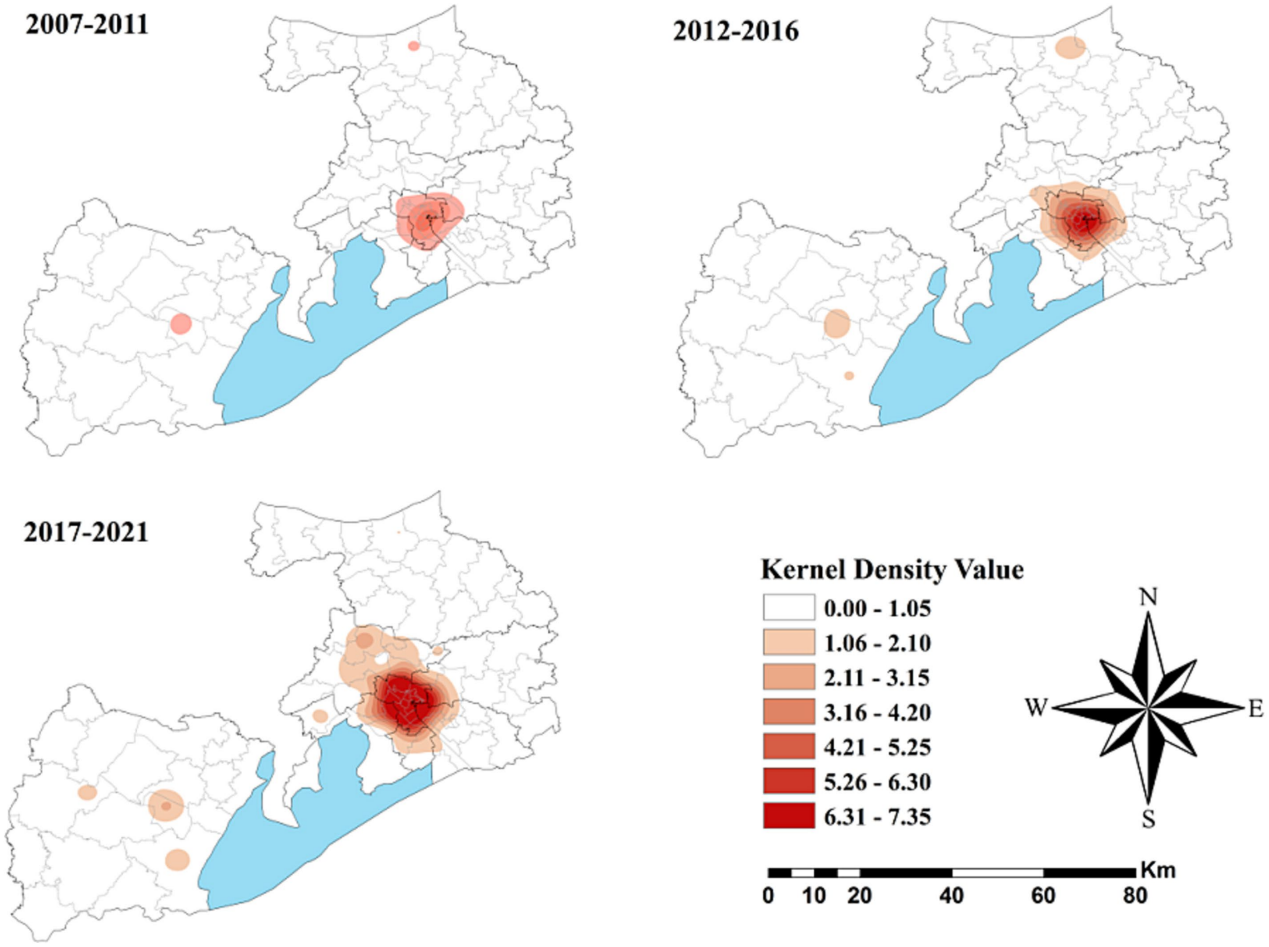
Spatial distribution of HCV cases in Wuxi City at the county level, 2007–2021.

Figure 4 depicts the spatial distribution of HCV cases in Wuxi City at the township level from 2007 to 2021, revealing pronounced spatial clustering in the central downtown area of Liangxi District. Comparing kernel density values across three time periods (2007–2011, 2012–2016, and 2017–2021) showed that high-density areas gradually expanded and that the values increased, particularly during 2017–2021. Similar spatial clustering patterns were also observed in Yixing, a county-level municipality located Southwest of Wuxi.

### 3.5 Cluster analysis

**Figure 5:**
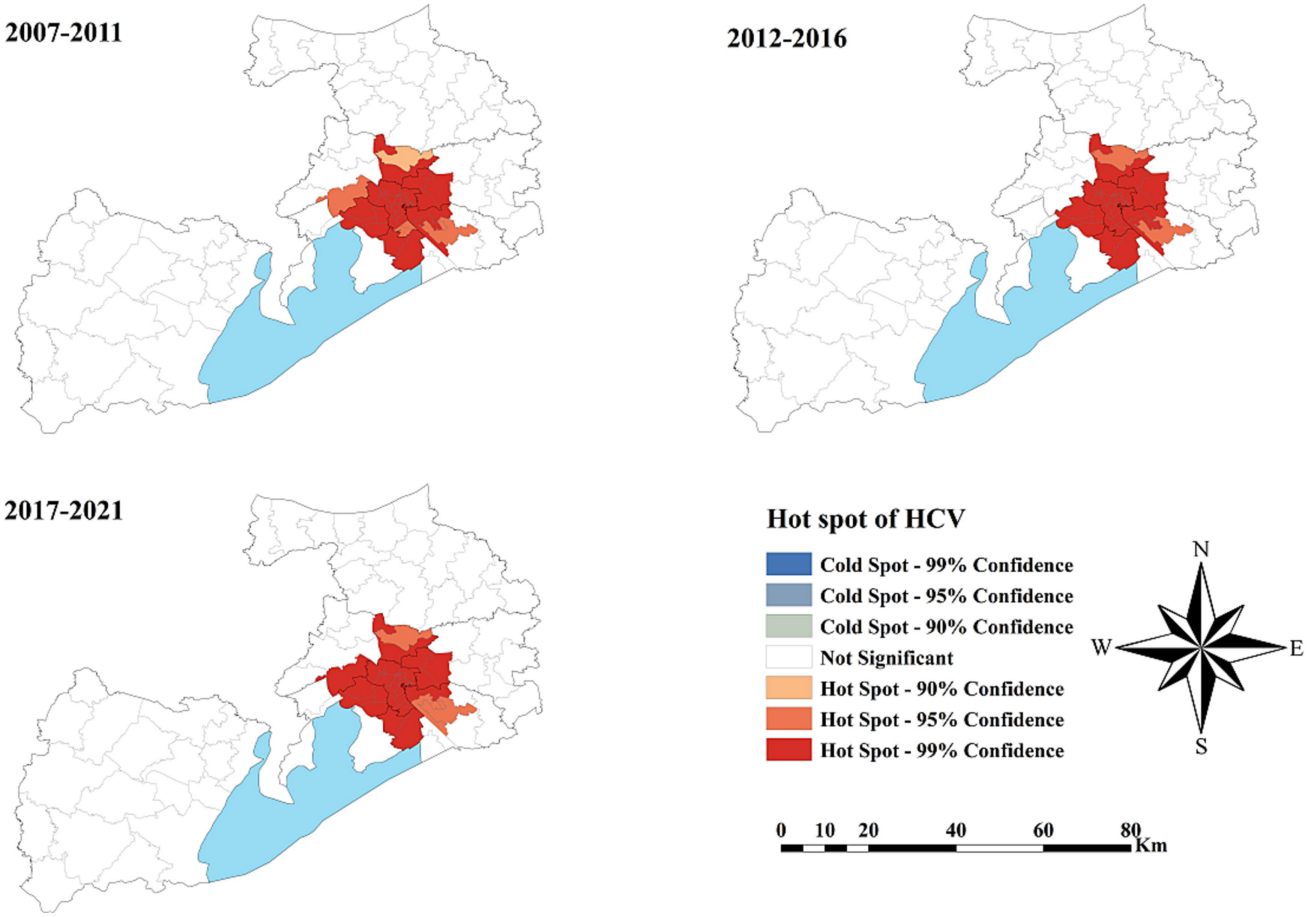
The spatial cluster of the hepatitis C virus incidence rate in Wuxi City at the county level, 2007–2021.

As shown in Figure 5, no statistically significant cold spots were observed during the study period. However, significant hot spots were consistently clustered in the Liangxi District and surrounding sub-districts across all three periods, with minimal spatial change over time.

## 4 Discussion

Since the identification of HCV in 1989, the Chinese government has implemented several measures to prevent and control its transmission (23). In the early decades, blood transfusion was the predominant transmission route of HCV in China. To address this, the National Health Commission of the People’s Republic of China (24) was enacted to strengthen supervision of blood collection and supply institutions. Beginning in 2015, HCV RNA screening became mandatory for all blood donations, including those from anti-HCV antibody-negative donors. Consequently, the dominant transmission route has shifted, and blood transfusion is no longer the primary contributor. Nonetheless, injecting drug use and unsafe sexual practices continue to facilitate the spread of HCV from high-risk groups to the general population, thereby contributing to the ongoing increase in HCV prevalence (15, 25).

Our study found that the average annual incidence rate of HCV infection in Wuxi City was 5.44 per 100,000 population, lower than the national average but higher than rates in Nanjing, Suzhou, and other cities in Jiangsu Province (26–30). From 2007 to 2021, the incidence in Wuxi City showed an overall upward trend, consistent with national and provincial data (26, 27). Several factors may explain this pattern. First, the upward trend likely reflects enhanced surveillance, greater awareness, and expanded screening over the 15-year period. To further curb viral hepatitis epidemics, two key policies were issued: “*China Viral Hepatitis Prevention and Control Plan (2017–2020)*” (31) and “*Action Programme to eliminate the Public Health hazards of Hepatitis C (2021–2030)*” (32). These programs emphasized “due inspection” of medical institutions and key groups, “willing inspection” for the general population, and “full coverage of nucleic acid testing” for HCV antibody-positive individuals (23), thereby improving case detection. Second, the gradual increase observed after 2017 was likely associated with the implementation of the latest version of the Diagnostic Criteria for Hepatitis C (WS 213–2018) (17) and improved reporting quality of infectious diseases in medical institutions. Under the updated criteria, individuals testing positive for anti-HCV antibodies must undergo nucleic acid testing, and only nucleic acid-positive individuals are reported as confirmed cases.

Our findings also indicated no significant seasonality in HCV incidence in Wuxi, consistent with previous studies (33–35). However, a sharp decline was observed in February 2020. This pattern may be explained by two factors. First, the “Spring Festival effect” identified in time-series analyses of notifiable diseases by Wei Shan at Fudan University (36), refers to reduced healthcare-seeking behavior during the Spring Festival period in February, when individuals prioritize family gatherings and travel. Second, during early 2020, strict COVID-19 control measures, including community/home isolation and traffic restrictions, substantially affected the reporting of notifiable infectious diseases (37).

Similar to other studies, the incidence rate of HCV infection was higher among males than in females (26–30). A study on HCV prevalence among Asian Americans in California also identified male sex as an independent risk factor for HCV infection (38). This difference may be attributed to the higher prevalence of risk behaviors among men, such as injection drug use and unprotected sexual activity. Age-group analysis showed that most cases occurred in individuals aged 40–59 and >60 years. This pattern can be explained by two factors. First, HCV is often asymptomatic for decades and may only progress to chronic disease later in life, creating a lag between infection and detection. Second, older adults have more frequent medical encounters, increasing their likelihood of undergoing HCV screening and passive detection.

Previous research has shown that genotype 3 is predominant in Southwest China (10). In our study, genotype 3 was the second most common in Wuxi City, possibly due to population mobility. As a highly developed city, Wuxi attracts migrant workers from underdeveloped regions, including Southwest China. Moreover, genotype 3 is more common among injecting drug users and individuals living with HIV infection (39, 40). Sentinel surveillance by the Jiangsu Provincial Centers for Disease Control and Prevention has shown that southern Jiangsu, including Wuxi, has higher populations of drug users and people living with HIV compared with northern Jiangsu (15). The distribution of HCV genotypes also varied by sex and age, possibly due to differences in behavioral and transmission routes among demographic groups.

Spatial distribution analysis revealed pronounced heterogeneity across Wuxi City during the 15-year study period. Kernel density mapping showed that HCV cases were mainly concentrated in the downtown area of Liangxi District, particularly during 2017–2021. The highest reported incidence was concentrated in the city center, possibly due to greater population density, active commercial activities, and a higher prevalence of invasive cosmetic procedures like tattooing and ear piercing, performed without adequate disinfection. Cluster analysis identified consistent hot spots in Liangxi District and surrounding sub-districts, but no significant cold spots. These stable hot spot patterns suggest that the underlying drivers of high HCV incidence in city centers have persisted over time (41). Establishing a four-component prevention and control system, comprising prevention, monitoring, screening, and treatment, should therefore be prioritized in key areas.

This study had certain limitations. First, the data were derived from reported cases; changes in diagnostic criteria for hepatitis C may have introduced selection bias. Second, incidence was calculated based on the resident population, which may underestimate infection rates in areas with large migrant populations. Future research should aim to obtain more accurate data on the floating population to refine estimates of HCV infection incidence and assess risk disparities between residents and migrants. Finally, socioeconomic status, healthcare accessibility, and testing policies were not included in the analysis, which may partly explain spatial clustering. Future studies should incorporate multivariable spatial modeling and relevant socioeconomic and healthcare covariates to better understand the determinants of HCV distribution.

## 5 Conclusion

This study identified the epidemiological characteristics, temporal trends, and spatial distribution of HCV infection in Wuxi City, a developed but low-epidemic area of eastern China. The incidence of HCV increased gradually between 2007 and 2021, with subtypes 1b and 3 as the most prevalent genotypes. Spatial clustering was concentrated in urban centers, which should be prioritized for targeted surveillance and intervention. Implementing focused prevention and control strategies will be essential to achieve the goal of eliminating HCV as a public health threat by 2030.

## Data Availability

The raw data supporting the conclusions of this article will be made available by the authors, without undue reservation.
